# Adaptive Hybrid Surgery: Paradigm Shift for Patient-centered Neurosurgery

**DOI:** 10.5041/RMMJ.10346

**Published:** 2018-07-30

**Authors:** Or Cohen-Inbar, Gil E Sviri

**Affiliations:** 1 Department of Neurological Surgery, Rambam Maimonides Health Care Campus, Faculty of Medicine, Technion–Israel Institute of Technology, Haifa, Israel; 2 Department of Neurological Surgery and Gamma-Knife Center, University of Virginia Health Care Campus, Charlottesville, VA, USA

**Keywords:** Adaptive hybrid surgery, planned subtotal resection, radiosurgery

## Abstract

The surgical management of cerebral and skull base lesions has evolved greatly in the last few decades. Still, a complete resection of lesions abutting critical neurovascular structures carries significant morbidity. Stereotactic radiosurgery (SRS) has emerged as an increasingly accepted treatment option. Minimally invasive, SRS results in excellent tumor control and low complication rates in patients with moderate-size tumors. The management of large cerebral and skull base tumors remains a formidable challenge. In such large tumors, radical surgical extirpation offers a significantly higher risk of neurological deficit, and SRS alone cannot be used because of the elevated incidence of radiation-induced complications known to be associated with large-volume tumors. With increasing treatment volumes, SRS-associated tumor control rates decrease and complication rates increase. Planned subtotal resection (STR) with adjuvant SRS (adaptive hybrid surgery [AHS]) has gained increasing interest in recent years as a multimodal approach. In AHS, a planned STR (aimed at decreasing surgical morbidity) followed by SRS to a preplanned residual tumor aids in harnessing advantages offered by both approaches. Although intuitive and reasonable, this paradigm shift from maximal resection at all cost has not been adopted widely. Combining open microsurgery with SRS requires a good understanding of both surgical and SRS modalities and their respective safety–efficacy features. We present a review and discussion on AHS as a modern, multidisciplinary treatment approach. Available data and views are discussed for vestibular schwannoma (VS) as a sample tumor. Other indications for AHS are mentioned in brief.

## INTRODUCTION

For many years, microsurgical extirpation has been considered a mainstay treatment option for different cerebral neoplastic lesions. One pivotal measure of success was whether or not the surgeon was able to achieve a gross total resection (GTR). This perception of neurosurgery is misleading, based on two assumptions, neither of which is true in modern times. The first assumption deals with the perception that the surgical intervention is surgeon-centered rather than patient-centered. The second assumes that microsurgical resection is the only available treatment option in the neurosurgeon’s armamentarium.

Microsurgical resection (i.e. GTR) has been regarded the treatment of choice for many cerebral and skull base tumors for decades. The surgical resection offers several advantages, such as histologic confirmation, relief of mass effect, and local compression imposed on adjacent neurovascular structures. In GTR for benign lesions, surgery offers in addition the possibility of cure.^1^ The surgical management of cerebral and skull base tumors has progressed significantly in recent years, in close correlation with advances made in microsurgical equipment and techniques, which have aided in reducing the morbidity associated with extirpation of these tumors.^2^–^4^ And still, a common dogma of skull based microsurgery acknowledges the concept that 95% of possible surgical complications occur upon tenacious resection of the final 5% of the tumor. This raises conceptual questions about the role of GTR.

Since its inception in the early 1950–1960s, stereotactic radiosurgery (SRS) has emerged as an increasingly accepted treatment option for patients with different intracranial pathologies.^1^,^5^–^17^ This technology has evolved dramatically since then (both software- and hardware-related quantum leaps), but its core principles remain unchanged. Ionizing radiation utilized in SRS is commonly gamma radiation (emitted by the radioactive cobalt [Co] isotope 60) or X-rays from linear accelerators (LINAC, Cyber-Knife, etc.). Stereotactic radiosurgery is designed and conceived with the aim of targeting and damaging intracranial targets through the intact skull, utilizing many highly focused beams of ionizing radiation. This is performed with the aid of stereotactic principles and image guidance. Each beam by itself carries very low quanta of radiation dose, yet the target volume at which the beams intersect receives a summated dose of radiation. Surrounding normal brain tissue receives insignificant levels of collateral radiation owing to the steep dose fall-off character of SRS.^1^

Stereotactic radiosurgery is considered a minimally invasive technique with exceedingly favorable tumor control rates and complication rates in patients with up to moderate-sized tumors of different histopathological origins. Several SRS reports of large series describing the treatment of small- to moderate-volume benign skull base lesions have reported more than adequate long-term tumor control rates coupled with impressively low related neurological morbidity and a good preservation of functions, compared with GTR.^1^,^5^,^7^–^10^,^15^–^22^

The management of large (typically defined as >13 mL in volume or 3 cm in largest diameter) cerebral and skull base tumors remains a formidable challenge. In such large tumors, radical extirpation as a sole approach yields unacceptable risks for neurological deficit, and SRS cannot be utilized as a first-line approach due to the elevated risk for radiation-induced complications associated with large-volume targets. With increasing treatment volumes, SRS-associated tumor control rates decrease and complication rates increase.^23^–^25^

**Figure 1:**
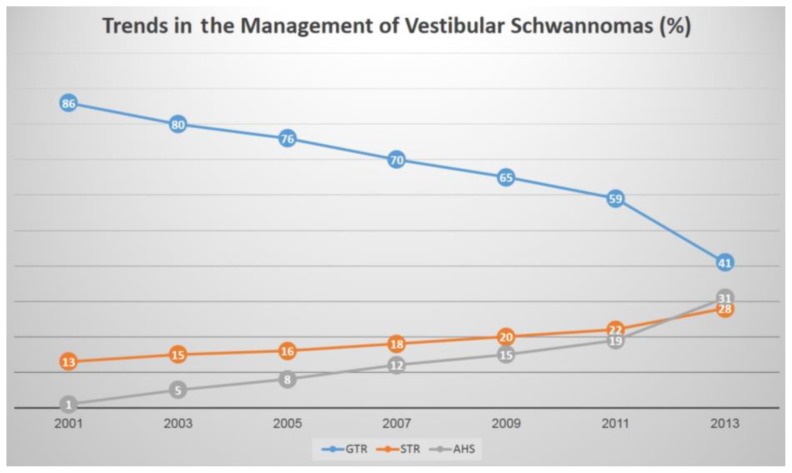
Changing Trends in the Clinical Management of Vestibular Schwannomas AHS, adaptive hybrid surgery; GTR, gross total resection; STR, subtotal resection. Refer to text.

Planned subtotal resection (STR) with adjuvant SRS approaches has gained increasing interest in recent years (Figure 1).^26^–^30^ Such shifting trends are evident in the management of vestibular schwannomas (VS), for example, in the United States in recent years,^27^,^28^ with an increasing number of surgeons aiming at a planned STR to decrease surgical morbidity and common surgical complications. The planned residual tumor (of smaller volume) is then approached radiosurgically, thus harnessing advantages offered by both approaches. Although intuitive and reasonable, this paradigm shift has not been adopted widely, neither practically nor conceptually. Furthermore, there is not much evidence to support a surgeon’s ability to decide during resection when the STR is both safe and sufficient, as discussed next.

Combining open microsurgery with SRS requires a good understanding of both treatment modalities and their respective efficacy–safety features. The microsurgeon needs to avoid direct manipulation and dissection between crucial structures and adjacent tumor capsule, even at the expense of leaving tumor tissue behind, to improve functional outcome. Thus, a “nerve-centered” or “crucial structures”-centered tumor surgery approach is needed on the one hand. The SRS surgeon, on the other hand, needs to accept and prepare for the fact that treatment planning and postsurgical debulking may be more challenging. This is largely due to modification of tumor bed local conditions, scarring, and debris as well as local scarring because of surgery, all confounding features when planning SRS target volume. Planning the extent and particulars for both the STR and the SRS procedures, including effective target delineation and timing after surgery, are key features to the success of this approach.^31^

We present a review and discussion on this concept of planned STR followed by SRS—at times referred to as adaptive hybrid surgery (AHS)—as a modern, multidisciplinary treatment approach. Available data and views are discussed for vestibular schwannoma (VS) as a sample tumor. Other indications for AHS are mentioned in brief.

## THE VESTIBULAR SCHWANNOMA STORY

Vestibular schwannomas (VS)—also known as acoustic neuromas—are common benign cerebellopontine angle neoplasms, arising from the vestibular portion of the eighth cranial nerve (CN). Vestibular schwannomas occur with an incidence of 1:100,000 person-years.^23^,^32^,^33^ Vestibular schwannoma typically presents with ipsilateral hearing deterioration (affects 95% of patients), and tinnitus (affects 60% of patients). Additional presenting symptoms include dizziness, vertigo, trigeminal neuropathy (12%), facial neuropathy (5%), and rarely caudal cranial nerve involvement (IX–XII).^34^

Management of VS aims at eliminating brainstem compression and cranial nerve palsies secondary to tumor encroachment, with no functional worsening. Treatment options include observation (“wait and scan”), microsurgical resection through different avenues (discussed next), SRS, and fractionated conventional radiotherapy (FRT). Treatment is advocated in general, for those symptomatic or showing rapid tumor volume growths, regardless of age or comorbidities. A growth rate of more than 2.5 mm/year correlates to lower hearing preservation rates. In addition, conservative management is known to correlate to progressive hearing loss, averaging 2.77–5.39 dB/year.^10^

For nearly a century, surgical extirpation was the only option available for patients with VS. Technical and technique-related improvements over time resulted in dramatic improvements in surgical operative outcome measures, yet a GTR is still associated with a significant risk of related complications.^35^ The retrosigmoid surgical approach is associated with higher preservation rates of both hearing and facial nerve function for VS >1.5 cm in largest diameter. Still, this approach harbors higher rates of postoperative cerebrospinal fluid (CSF) leaks and headaches. The middle fossa surgical approach offers better outcomes for those with VS <1.5 cm in largest diameter. The translabyrinthine surgical approach in turn is reserved for patients with no functional hearing.^10^

Treatment of VS with SRS offers superior tumor control and facial nerve function preservation rates, with comparable hearing preservation rates. Current evidence supports the claim that SRS is associated with higher hearing and facial nerve function preservation rates and overall better functional outcomes. This is achieved with similar tumor control rates compared to microsurgical approaches. Stereotactic radiosurgery is currently considered the treatment of choice for VS <3 cm in largest diameter.^10^ For larger VS (>3 cm or 13 mL in volume), AHS should be considered as a safer option, with surgical debulking followed by SRS for the residual tumor as discussed next.

### Tumor Volume Control

There is much controversy over complete versus incomplete removal of VS as the best management strategy. On the one hand, there is a clear proven correlation between the postoperative residual tumor volume and risk of progression or recurrence.^31^ A recent report by Vakilian et al.^36^ further supports this claim. A VS postsurgical volume >2.5 cm^3^ showed recurrence in all cases in their report.^36^ Reported rates of postsurgical facial nerve damage and hearing dysfunction remain significant.^37^–^40^ On the other hand, tumor control rates of SRS (gamma-knife or LINAC-based) for small and medium-size VS are reported to reach as much as 97.5% of cases, combined with a 97% rate of facial nerve functional preservation and a median size decrease of 40% at 7 years post-SRS follow-up.^10^,^41^ Thus, SRS allows for acceptable functional results in moderate-volume tumors and has emerged as the preferred upfront treatment alternative in such cases.^19^,^20^,^22^

With the advances being made in all fields of medicine that improve quality of life, patients with large VS have higher expectations and lower tolerance and acceptance with regard to the functional outcome of VS; they expect and want similar functional outcomes as for those undergoing SRS for smaller-volume tumors in terms of neurological outcome and postoperative deficits. Tolerance for cosmetic and other complications such as facial nerve palsy has decreased dramatically. Such a complication is considered unacceptable nowadays and should be avoided at all costs.^23^ To date, reports on outcome for AHS (subtotal removal followed by adjuvant SRS) for larger-volume VS have been scarce and cohort sizes limited.^42^–^48^

In a recent meta-analysis by Rykaczewski et al.^49^ on SRS for VS (including AHS), which encompassed 28 studies (2007–2011) and 3,233 patients, mean tumor control of 92.7% was reported, at an average follow-up of 51.24 months.^49^ There is further support to the concept^23^,^50^,^51^ that AHS provides both excellent tumor control rates and desired preservation of facial nerve function.^42^–^48^,^52^,^53^

### Facial Nerve Preservation

In microsurgery of large VS, the size of the tumor preoperatively serves as the key predictor for facial nerve preservation, both anatomically and functionally.^4^,^38^ The incidence of surgically induced facial nerve palsy in those with VS >3 cm is 6-fold greater than in those with smaller lesions.^54^

Reviewing available studies reporting a cranial nerve morbidity measure for those who received GTR versus STR for large-volume VS returns mixed results.^29^,^55^ Of note, most of the surgical series reporting postoperative facial nerve morbidity consider patients with House–Brackmann (HB) I–III as good results. However, from a functional and cosmetic point of view as well as quality of life, HB II–III is by no means normal function.^31^ Still, these results suggest that although surgeons prefer safety over GTR, STR in current practice (random, not as part of AHS) can still cause undue neurologic deficits without the added gain of GTR.^31^ However, STR of large VS has been reported to achieve excellent facial nerve function preservation rates in 80%–100% and serviceable (functional) hearing in up to 100% of patients.^48^ Pollock et al. performed a meta-analysis and compared postoperative facial nerve palsy after microsurgery and SRS, reporting it to be 19% and 1%, respectively.^56^

Although the influence of extent of resection on postoperative facial nerve preservation remains a matter of debate, the concept that keeping intra-operative mechanical stress during STR to a minimum might help reduce neural morbidity appears to be more widely accepted.^23^ Accordingly, even when facial nerve electrophysiological monitoring is used (as in most cases), some argue for termination of surgery before the facial nerve stimulation signal is lost.^57^ However, since the surgeon cannot accurately predict when this will happen,^58^,^59^ many argue for leaving tumor tissue abutting the nerve if the need arises. Better postprocedural facial nerve preservation rates have been achieved by AHS with STR, in the range of 82%–100% (HB I–II).^42^,^46^,^60^–^62^

### Adaptive Hybrid Surgery for Large VS

One can approach STR for large VS using two different strategies. One consists of a planned STR in which the surgeon directs the surgical effort to preserve the cranial nerve and brainstem, resecting only the volume of tumor necessary for converting the residual volume to an ideal SRS target. This approach yields the best combined outcome, as recently reviewed by Iwai et al.^53^ and Daniel et al.^31^ A second approach consists of performing an STR (near-GTR), geared at leaving as little tumor as possible, which typically occurs at the level of the internal acoustic meatus. Since the facial nerve is particularly vulnerable at this location, this strategy has proven less favorable. The latter approach does not represent a real AHS approach.

Jeltema et al.^63^ reported a series of large-VS patients in which microsurgery was aimed at near-GTR with salvage SRS only when the residual volume showed growth. The authors reported normal facial nerve function (HB I) in 57.7%. In this study, 32.7% had mild (HB II and III) postoperative facial function dysfunction, and 9.6% had severe (HB IV and V) palsies. As discussed, this is not a true AHS approach, and most would argue for leaving a larger residual tumor in place to be followed by planned SRS. Concerning the timing of SRS after planned STR (as part of AHS), when larger VS residuals are left in surgery, many argue that complementary SRS should be performed in the months following surgery.^31^,^53^

Pan et al.^62^ compared two treatment approaches for large VS: AHS (group 1, *n*=18) versus GTR (group 2, *n*=17). Excellent facial nerve functional outcome (HB I and II) was noted in 89% and 35% in groups I and II, respectively. Hearing preservation was 100% and 0% in groups I and II respectively. Similar results were reported by Van de Langenberg et al.^46^ in a series of 50 patients. Brokinkel et al.^50^ published a recent systematic review analyzing six studies of AHS (gamma-knife radiosurgery following STR). A cohort of 159 patients with tumor diameters greater than 2 cm were reviewed. The average follow-up was 15 months, in which time excellent facial nerve function (HB I and II) was noted in 94%, and serviceable (functional) hearing preserved in 11.6%.

Daniel et al.^31^ reviewed 32 patients treated with AHS. Median follow-up was 24 months (range 4–78). Average presurgical tumor volume was 12.5 mL (range 1.47–34.9). Of note, in their series, Daniel et al.^31^ controlled the extent of surgical resection and capsular excision, progressing only until both superior and inferior borders of the facial nerve were identified surgically with electrophysiological neuro-monitoring. Thus, in a much more conservative surgical approach, a thin cuff of tumor capsule around the facial nerve course was intentionally left behind.^31^

### Adaptive Hybrid Surgery Software

**Figure 2:**
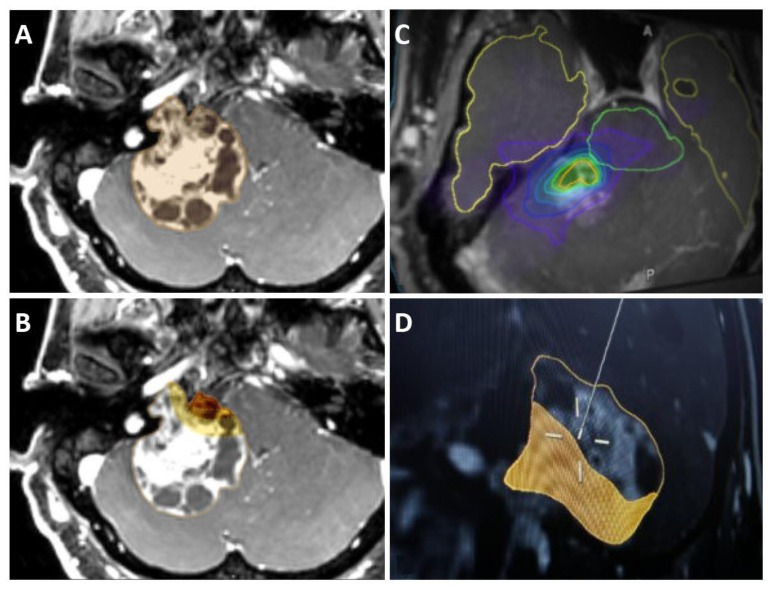
Adaptive Hybrid Surgery Software (with Permission, from Brainlab Inc.) **A)** T1WI MRI showing a large right vestibular schwannoma abutting brainstem and adjacent structures. **B)** Planning STR, objective criteria for extent of STR. **C)** Simulation and optimization of residual tumor for SRS, radiation plan. **D)** Intraoperative update option for adjuvant SRS plan.^65^

The adaptive hybrid surgery software (Brainlab AG, Munich, Germany(, approved by the US Food and Drug Administration in 2014,^64^ simulates treatment plans (SRS, FRT) for postsurgical tumor volumes. The software assists in defining an SRS-ideal residual tumor volume (Figure 2).

The AHS software allows the surgeon to define a realistic residual tumor target. Next, it formulates three separate radiation plans (possible approaches) for each tumor volume predefined by the surgeon for different complementary radiation tools (SRS, hypofractionated SRS, and FRT). The AHS software helps in balancing the risks of microsurgery (based on the surgeon’s initial delineation of a realistic residual tumor volume) against the risks of radiation-related toxicity to critical adjacent neurovascular structures from the SRS.^65^

Yang and his co-authors^66^ retrospectively compared target volume delineation defined manually by a surgeon to those formulated by the AHS software in seven patients with VS. The planned volumes were significantly smaller in the manual schemes as compared to those offered by the AHS software (1.6 mL versus 4.5 mL, *P*=0.004). The mean residual volumes were significantly smaller than the ideal volumes defined by AHS (2.2 mL versus 4.5 mL; *P*=0.02). As discussed, a smaller postsurgical residual volume translates to higher facial nerve damage rates and hearing loss.

## ADDITIONAL INDICATIONS FOR AHS

### Skull Base Meningioma

Meningiomas are common, typically benign, intracranial extra-axial tumors, constituting 12%–20% of primary intracranial tumors. Most are benign WHO-I lesions, but their treatment can still pose a formidable challenge. Gross total resection is often not achieved nor planned due to tumor proximity to pivotal neurovascular structures. This often leads to higher recurrence rates and residual tumor progression.^15^,^67^–^69^ One such challenging location is the parasellar region. Meningiomas in this location tend to invade adjacent suprasellar, cavernous sinus, and petroclival regions, at times involving crucial neurovascular structures (internal carotid artery and branches, cranial nerves, etc.).^8^,^15^,^70^,^71^ Stereotactic radiosurgery is an important complementary treatment option (both in the frame of AHS and as upfront approach) in managing such inaccessible, recurrent, or residual lesions.^1^ Stereotactic radiosurgery has emerged as a minimally invasive and durable treatment option for these meningiomas, offering a combined favorable profile of high tumor control rates and a low incidence of neurological deficits compared with other treatment options.^8^

### Pituitary Adenomas

Pituitary adenomas are a very common type of intracranial tumor (10%–20% of intracranial tumors).^7^ While their anatomical location and macroscopic appearance are relatively similar, such tumors differ widely: functioning versus non-functioning adenomas, secretory versus non-secretory adenomas, locally aggressive or not, treatment-responsive or resistant, etc. Hence, treatment options vary and include hormonesuppressive medical therapy, microscopic/endoscopic resection, SRS, FRT, or observation.

Some pituitary adenomas spread locally to invade surrounding meninges (dura) and cavernous sinus, hampering a complete surgical resection.^5^,^16^,^17^ Stereotactic radiosurgery is commonly utilized as an adjunct following incomplete surgical resection (as part of an AHS approach), tumor recurrence, or medical therapy failure.^7^ Those with a residual functioning pituitary adenoma causing acromegaly or Cushing’s disease after resection require AHS as a routine approach in order to achieve durable tumor control rates.^16^ Silent corticotroph-staining adenomas are a rare subset of non-functioning adenomas shown to be more locally aggressive, requiring AHS as a routine approach in order to achieve durable tumor control rates.^5^ Prolactin-secreting adenomas are a common type of functioning adenomas, most effectively treated with dopamine agonists. A minority of patients do not respond to medications (dopamine agonist-resistant prolactinomas) or are intolerant to these owing to related side effects. Microsurgical resection, when feasible, is the next option, yet cavernous sinus, dural, or bone invasion/involvement may preclude a complete resection, in which case an AHS approach should be adopted.^17^

### Non-vestibular Schwannomas

To date, GTR has been a preferred treatment for non-vestibular schwannomas. This group of lesions refers to intracranial schwannomas arising from other cranial nerves (i.e. non-CN-VIII), such as trigeminal/Meckel’s cave schwannomas and jugular foramen schwannomas (arising from CN IX–XI). These tumors are typically benign (WHO-I).

Although GTR is potentially curative and desirable, it is almost always impossible to achieve a GTR without related complications, because of proximity to abutting adjacent critical neurovascular structures. Stereotactic radiosurgery provides acceptable tumor control rates with much lower related morbidity compared to microsurgery for smaller lesions.^72^ An AHS approach is advocated for larger lesions.^73^

## CONCLUSION

A combined strategy of planned STR and scheduled postoperative SRS for the postsurgical residual tumor provides patients with the desired combination of exceedingly high tumor control rates and favorable clinical outcome (preservation of neurological function and quality of life). As the cranial nerve morbidity of GTR may be unacceptably high, and the tumor control rate for small lesions treated with SRS is likewise high, it stands to reason that patients with large tumors be managed with the AHS approach (planned STR and adjuvant SRS). Such patients, with tumors too large to be treated safely solely with SRS, require a pre-SRS surgical decompression. The surgical goal in such instances is to decompress the normal structures and to create an ideal target for a future SRS treatment.

To clarify, we do not advocate suboptimal surgical results by leaving large-volume residual tumors in patients undergoing surgery. However, the primary goal of treatment is the patient’s overall benefit. Hence, we see the goal of surgery to be tumor debulking and preservation of neurological status. Larger residual postoperative volumes, that are small enough to allow for a safe and effective SRS, may be an appropriate compromise in order to achieve this desired clinical outcome. Standardized reproducible planning tools are mandated, allowing assessment of different AHS approaches and comparison of different techniques in order to advance this technique, for patient safety and health.

## Abbreviations

AHS: adaptive hybrid surgery
CN: cranial nerves
CSF: cerebrospinal fluid
GTR: gross total resection
HB: House–Brackmann grade
LINAC: linear accelerator
SRS: stereotactic radiosurgery
STR: subtotal resection
VS: vestibular schwannoma.
